# Supplementary figures and images for: Proinflammatory Risk Factors in Patients with Ischemic Stroke: A Systematic Review and Meta-Analysis
Source: Antioxidants (Basel). 2025 Oct 14;14(10):1229. doi: 10.3390/antiox14101229 (PMC12561542; doi:10.3390/antiox14101229)

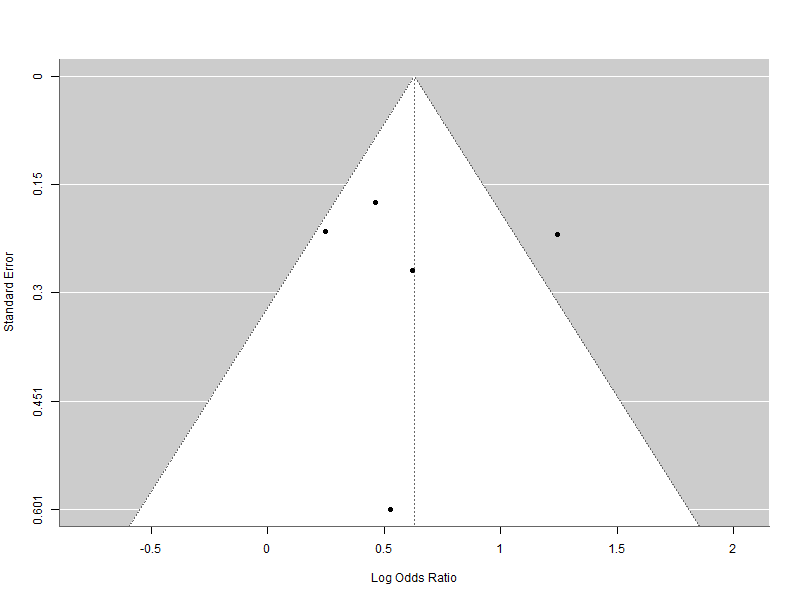

Supplement: Supplementary file 1 [file antioxidants-14-01229-s001.zip › funnel_plot_atrial_fibrillation.png]

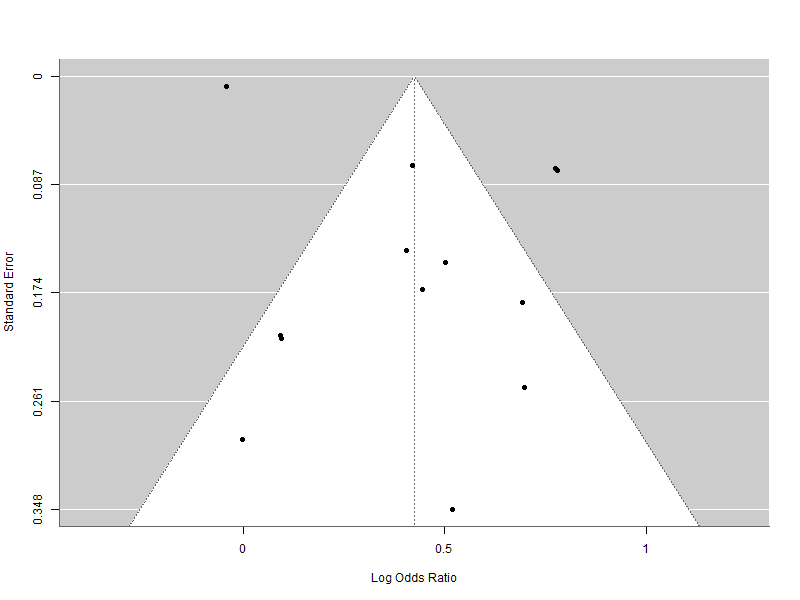

Supplement: Supplementary file 1 [file antioxidants-14-01229-s001.zip › funnel_plot_diabetes.png]

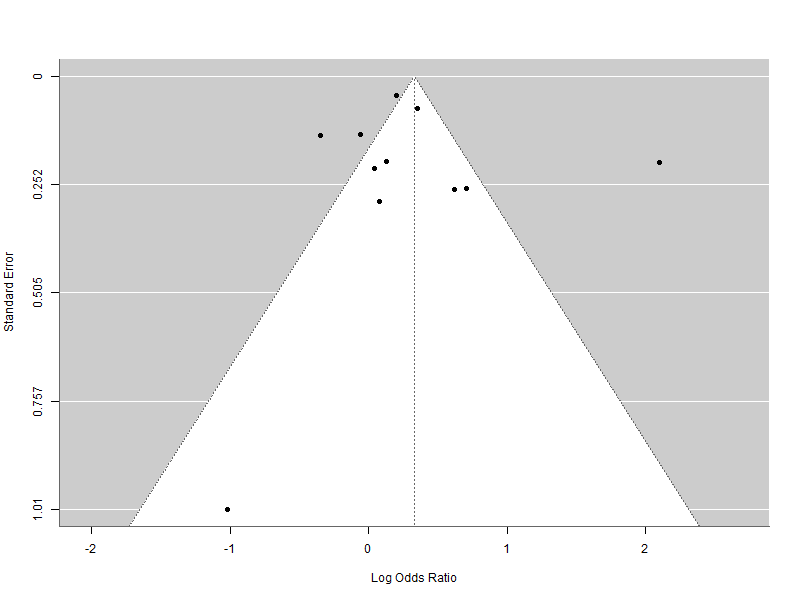

Supplement: Supplementary file 1 [file antioxidants-14-01229-s001.zip › funnel_plot_dyslipidemia.png]

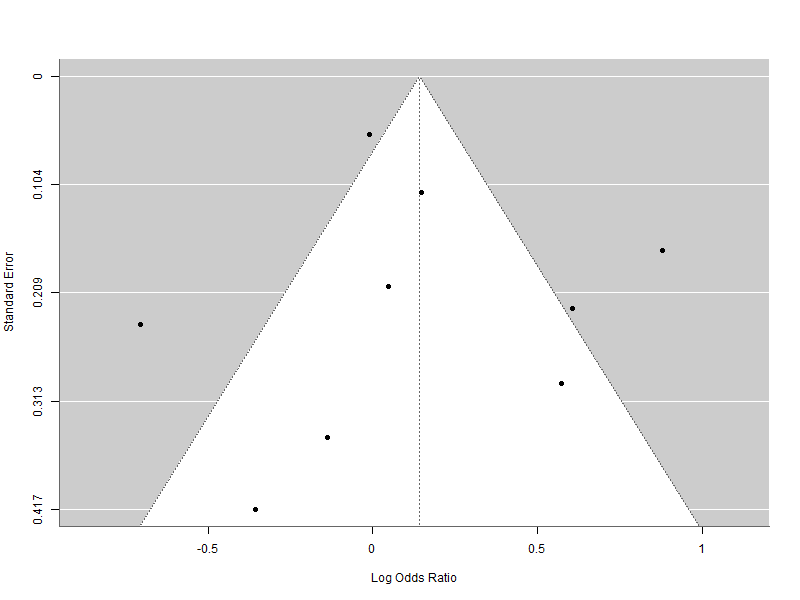

Supplement: Supplementary file 1 [file antioxidants-14-01229-s001.zip › funnel_plot_factorX.png]

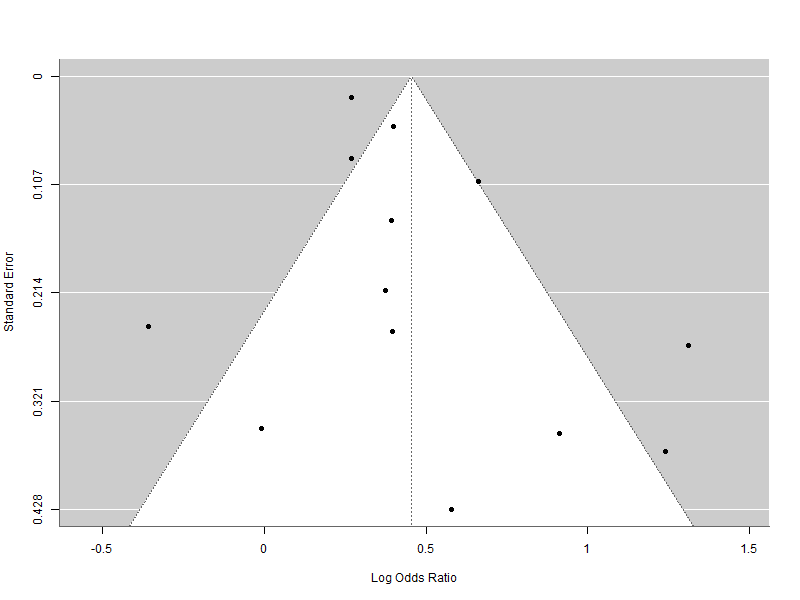

Supplement: Supplementary file 1 [file antioxidants-14-01229-s001.zip › funnel_plot_hypertension.png]

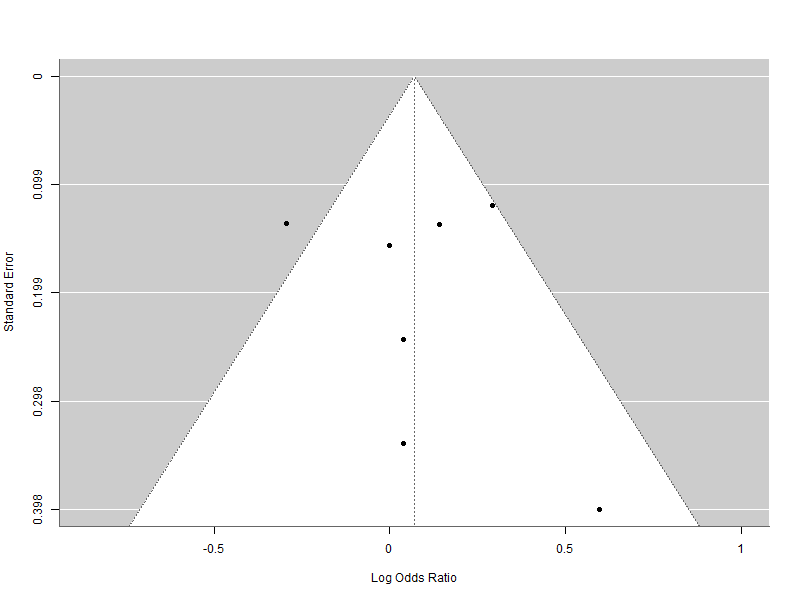

Supplement: Supplementary file 1 [file antioxidants-14-01229-s001.zip › funnel_plot_obesity.png]

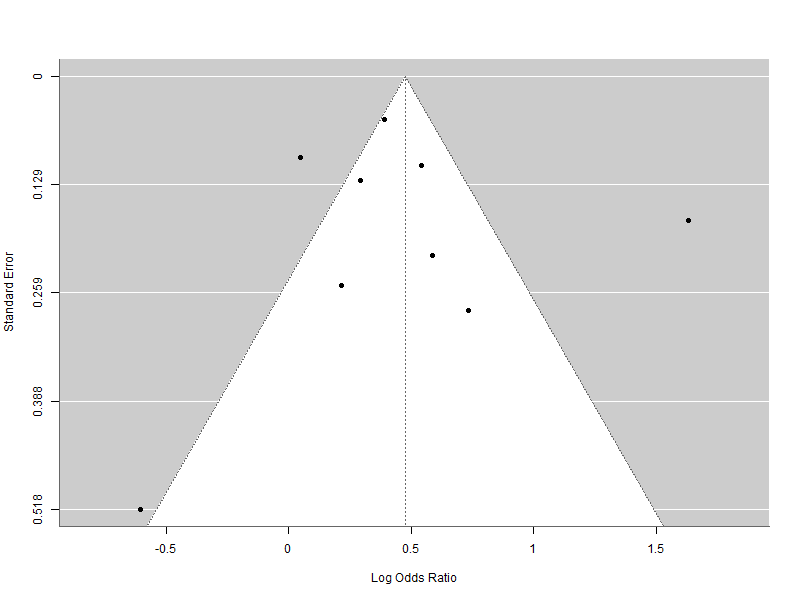

Supplement: Supplementary file 1 [file antioxidants-14-01229-s001.zip › funnel_plot_smoking.png]

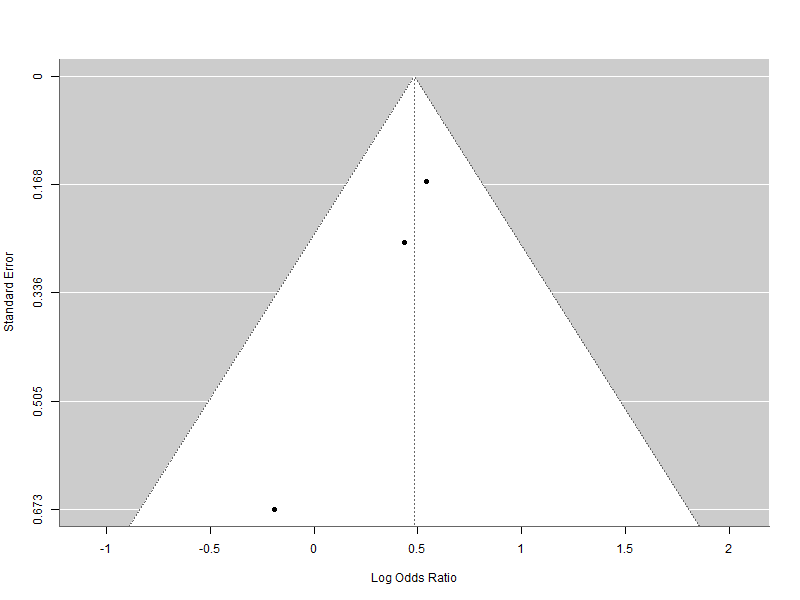

Supplement: Supplementary file 1 [file antioxidants-14-01229-s001.zip › funnel_plot_TIA.png]
